# SZC-6 Promotes Diabetic Wound Healing in Mice by Modulating the M1/M2 Macrophage Ratio and Inhibiting the MyD88/NF-χB Pathway

**DOI:** 10.3390/ph18081143

**Published:** 2025-07-31

**Authors:** Ang Xuan, Meng Liu, Lingli Zhang, Guoqing Lu, Hao Liu, Lishan Zheng, Juan Shen, Yong Zou, Shengyao Zhi

**Affiliations:** 1 Guangdong Provincial Key Laboratory of Pharmaceutical Bioactive Substances, Guangdong Pharmaceutical University, Guangzhou 510006, China; 2112255040@gdpu.edu.cn (A.X.); 2112255038@gdpu.edu.cn (M.L.); 2112240203@gdpu.edu.cn (L.Z.); 2112355032@gdpu.edu.cn (L.Z.); shenjuan@gdpu.edu.cn (J.S.); 2 School of Basic Medicine, Guangdong Pharmaceutical University, Guangzhou 510006, China; 3 State Key Laboratory of Anti-Infective Drug Discovery and Development, School of Pharmaceutical Sciences, Sun Yat-Sen University, Guangzhou 510006, China; luguoqing@mail.sysu.edu.cn; 4 School of Nursing, Department of Nursing, Foshan Fetal Medicine Research Institute, Affiliated Foshan Maternity & Child Healthcare Hospital, Southern Medical University, Foshan 528100, China; liuhao@gdpu.edu.cn; 5 School of Life Sciences and Biopharmaceutics, Guangdong Pharmaceutical University, Guangzhou 510006, China

**Keywords:** diabetic, SZC-6, macrophages, wound healing

## Abstract

**Background/Objectives**: The prolonged M1-like pro-inflammatory polarization of macrophages is a key factor in the delayed healing of diabetic ulcers (DU). SIRT3, a primary mitochondrial deacetylase, has been identified as a regulator of inflammation and represents a promising new therapeutic target for DU treatment. Nonetheless, the efficacy of existing SIRT3 agonists remains suboptimal. **Methods**: Here, we introduce a novel compound, SZC-6, demonstrating promising activity levels. **Results**: SZC-6 treatment down-regulated the expression of inflammatory factors in LPS-treated RAW264.7 cells and reduced the proportion of M1 macrophages. Mitosox, IF, and JC-1 staining revealed that SZC-6 preserved cellular mitochondrial homeostasis and reduced the accumulation of reactive oxygen species. In vivo experiments demonstrated that SZC-6 treatment accelerated wound healing in diabetic mice. Furthermore, HE and Masson staining revealed increased neovascularization at the wound site with SZC-6 treatment. Tissue immunofluorescence results indicated that SZC-6 effectively decreased the proportion of M1-like cells and increased the proportion of M2-like cells at the wound site. We also found that SZC-6 significantly reduced MyD88, p-IκBα, and NF-χB p65 protein levels and inhibited the nuclear translocation of P65 in LPS-treated cells. **Conclusions**: The study concluded that SZC-6 inhibited the activation of the NF-χB pathway, thereby reducing the inflammatory response and promoting skin healing in diabetic ulcers. SZC-6 shows promise as a small-molecule compound for promoting diabetic wound healing.

## 1. Introduction

Diabetes mellitus is a complex, multisystem, chronic metabolic disorder. According to the International Diabetes Federation, approximately 589 million adults aged 20–79 are projected to have diabetes by 2025, representing 11.1% of the global population in this age group [1]. Chronic hyperglycemia leads to severe complications, including cardiovascular, renal, retinal, and neurological disorders, which are the primary causes of diabetes-associated morbidity and mortality [2,3]. Among these, DU is one of the most prevalent and debilitating complications, characterized by chronic, non-healing wounds that impose substantial morbidity, mortality, and economic burdens [4,5]. Patients with DU are particularly susceptible to infections; without timely and effective intervention, ulcers may progress to gangrene, osteomyelitis, and often necessitate lower-limb amputation [6]. Current clinical interventions, such as systemic hyperbaric oxygen therapy (s-HBOT), platelet-rich plasma (PRP), and negative pressure wound therapy (NPWT), have demonstrated some efficacy but are limited by various drawbacks [7]. While platelet-rich plasma (PRP) accelerates wound healing, its efficacy as a monotherapy may be insufficient for preventing complications in high-risk patients, underscoring the necessity for novel therapeutic strategies.

DU pathogenesis involves impaired angiogenesis and dysregulated inflammatory responses [8,9,10]. The transition from inflammation to proliferation is a critical phase in wound healing [11]. However, persistent chronic inflammation driven by excessive cytokine production and immune cell infiltration often disrupts this process in DU [12]. Macrophages play a pivotal role in this context, where polarization toward the anti-inflammatory M2 phenotype facilitates wound healing [13]. In diabetic conditions, the inflammatory milieu impairs the M1-to-M2 macrophage transition, resulting in an increased M1/M2 ratio in affected skin tissues [14].

Recent studies have reported a decrease in Sirtuin 3 (SIRT3) expression in tissues of diabetic patients compared to healthy controls [15]. SIRT3 overexpression promotes M0-to-M2 macrophage polarization and mitigates inflammation and apoptosis, as demonstrated in bone marrow-derived macrophages (BMDMs) and glyoxylic acid-induced kidney stone (KS) mice [16]. Qin et al. further showed that SIRT3 upregulation suppresses NF-χB activation and promotes M2 polarization in RANKL-stimulated RAW264.7 macrophages in vitro [17]. Natural product-derived SIRT3 agonists, such as hesperidin (HST) and hepcidin, have shown potential in treating diabetic wounds and acute kidney injury by upregulating SIRT3 expression [18]. However, their clinical translation is hindered by poor solubility and suboptimal activity.

7-hydroxy-3-(4-methoxyphenyl)-2H-chromen-2-one (C12) was identified as the first SIRT3 agonist through structure-based design targeting the MnsodK68AcK-SIRT3 complex [19]. Building upon C12, we developed SZC-6, a modified 3-aryl coumarin derivative optimized for enhanced SIRT3 allosteric binding [20]. Given the reported anti-inflammatory effects of coumarin derivatives [21], we investigated whether SZC-6 exerts similar properties. Our study revealed that SZC-6 reduced reactive oxygen species (ROS) levels, restored mitochondrial membrane potential, decreased the proportion of M1 macrophages, and lowered inflammatory factor secretion. In vivo, SZC-6 treatment accelerated wound healing in diabetic mice by modulating the M1/M2 macrophage ratio. Our findings indicated that SZC-6 inhibited NF-χB pathway activation, thereby attenuating inflammation and promoting skin repair in DU. These findings introduce SZC-6 as a promising therapeutic candidate for diabetic ulcer management.

## 2. Results

### 2.1. SZC-6 Enhances SIRT3 Deacetylase Activity

**Figure 1:**
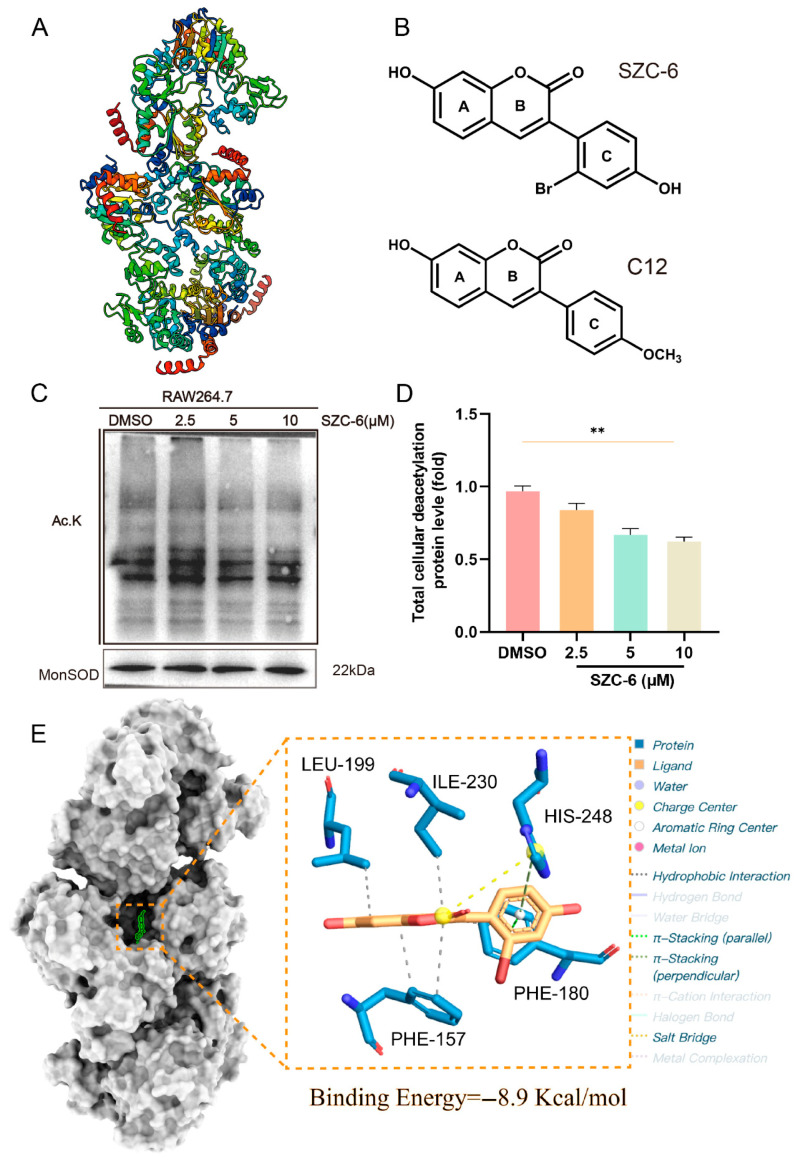
A selective SIRT3 activator, SZC-6, enhanced deacetylation activity. (**A**) Three-dimensional view of SIRT3 protein structure. (**B**) Chemical structure of SZC-6 and C12. (**C**,**D**) RAW264.7 cells were treated with various doses of SZC-6. Total cellular deacetylation was probed with an anti-acetyl lysine antibody to evaluatelysine acetylation. (**E**) The predicted co-crystal structure of SZC-6 and SIRT3. Left, overall structure with SIRT3 depicted in grey. Right, enlarged view of the isomeric site of SZC-6. SIRT3 is represented as a grey cartoon, while SZC-6 is illustrated as a rod. Dotted lines represent hydrogen bonds. Data are presented as mean ± SD (*n* = 3). ** p < 0.01.

Compared to orthosteric ligands, allosteric ligands display greater selectivity and improved physicochemical properties, enabling them to modulate challenging pharmacological targets effectively. The accurate identification of allosteric sites is crucial for the development of allosteric drugs. Notably, a pocket structure near Phe180 and Phe294 on the surface of SIRT3 has been identified as a potential target site for metastasis (Figure 1A) [22]. We synthesized a novel SIRT3 activator, SZC-6, by using a combination of computational and experimental techniques. This compound exhibits enhanced SIRT3 agonist activity and greater selectivity compared to C12 (Figure 1B) [20]. Furthermore, SZC-6 was applied at 2.5, 5, and 10 μM for 24 h, showing dose-dependent increases in cellular deacetylation using Western blot analysis. (Figure 1C,D). SIRT3 features a two-domain structure, which includes a binding site within a Rossmann fold domain that binds NAD+ with a zinc binding motif and a smaller domain with a helical bundle. Molecular docking analyses indicate that SZC-6 forms H-bonds with PHE180 and ILE230 of SIRT3, while their hydroxybenzene rings penetrate deeply into the cleft between the two structural domains, creating an aryl-aromatic stack with His248. This interaction is crucial for the deacetylation activity of SIRT3 (Figure 1E).

### 2.2. SZC-6 Improves Mitochondrial Function and Reduces ROS Levels

**Figure 2:**
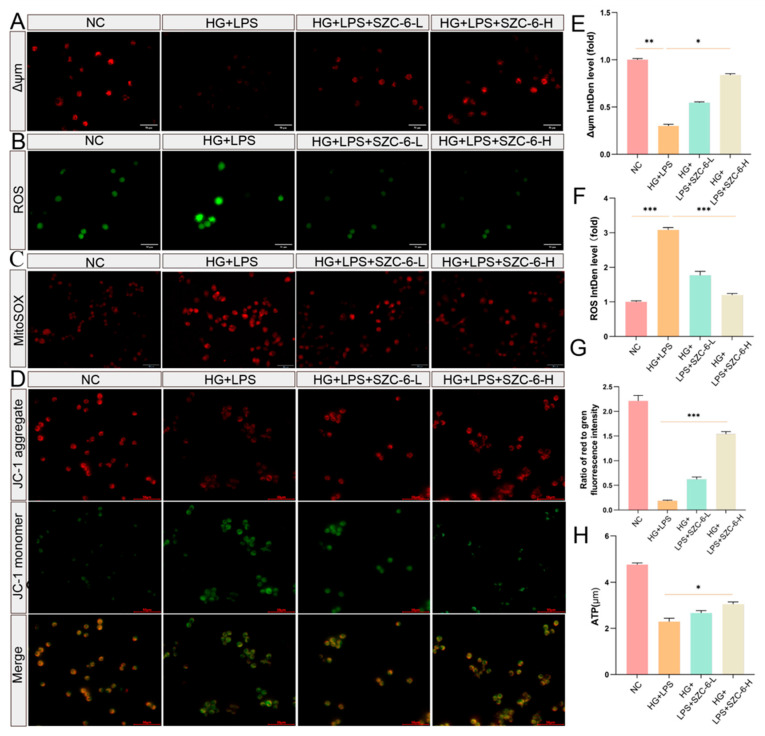
SZC-6 reduces ROS accumulation and increases mitochondrial potential in vitro. (**A**) Mitochondrial membrane potential (Δψm) of RAW264.7 was assessed using Image-iT™ TMRM dye after treatment with HG + LPS and HG + LPS + SZC-6. Scale bar: 50 μm. (**B**) Intracellular reactive oxygen species (ROS) content was measured using a ROS detection kit. Scale bar: 50 μm. (**C**) SZC-6 reduces intracellular mitochondrial ROS accumulation. Scale bar: 50 μm. (**D**) Mitochondrial membrane potential (Δψm) of RAW264.7 was measured with the JC-1 probe after treatment with HG + LPS and HG + LPS + SZC-6. Red and green fluorescence corresponded to JC-1 aggregates and monomers, respectively. Scale bar: 50 μm. (**E**,**F**) Statistical plots showing mitochondrial membrane potential (Δψm) and ROS fluorescence intensity. (**G**) Quantification of the red/green fluorescence ratio in (**D**). (**H**) Total intracellular ATP levels. Data are presented as mean ± SD (*n* = 3). * p < 0.05, ** p < 0.01, *** p < 0.001. NC refers to negative control, LPS refers to the high-glucose model group, SZC-6-L denotes 5 μM SZC-6, and SZC-6-H denotes 10 μM SZC-6.

In diabetic patients, reduced expression of SIRT3 has been associated with mitochondrial dysfunction and excessive ROS accumulation, resulting in impaired wound healing [15,23]. It is suggested that reducing excessive ROS accumulation may be a potentially effective treatment strategy for individuals with diabetic foot ulcers. A hyperglycemic inflammatory model was established using high glucose (30 mmol/L) plus LPS (1 μg/mL), followed by co-incubation with SZC-6-L (5 μM) or SZC-6-H (10 μM) for 24 h. Our results indicated that both low and high doses of SZC-6 increased mitochondrial membrane potential and decreased intracellular ROS accumulation compared to the LPS model group (Figure 2A,B). ROS accumulation staining indicated that SZC-6 treatment reduced intracellular mitochondrial reactive oxygen species accumulation (Figure 2C). We also assessed the mitochondrial response to inflammation levels using JC-1 staining. The LPS group exhibited higher green fluorescence intensity than the NC group, indicating decreased mitochondrial membrane potential (Δψm). Conversely, SZC-6 treatment reduced green fluorescence intensity and increased red fluorescence intensity, suggesting improved mitochondrial membrane potential (Δψm) (Figure 2D). Quantitative analysis of Δψm, ROS fluorescence intensity, and JC-1 staining indicated stronger therapeutic effects in the high-dose group (Figure 2E–G). Additionally, we measured the total intracellular ATP levels and found that SZC-6 increased ATP levels within the LPS group (Figure 2H). In summary, these results suggest that SZC-6 increases the total ATP levels and mitochondrial membrane potential, reduces intracellular ROS accumulation, and improves oxidative stress conditions.

### 2.3. SZC-6 Improves Mitochondrial Fusion and Fission in a High-Glucose Inflammatory Context

**Figure 3:**
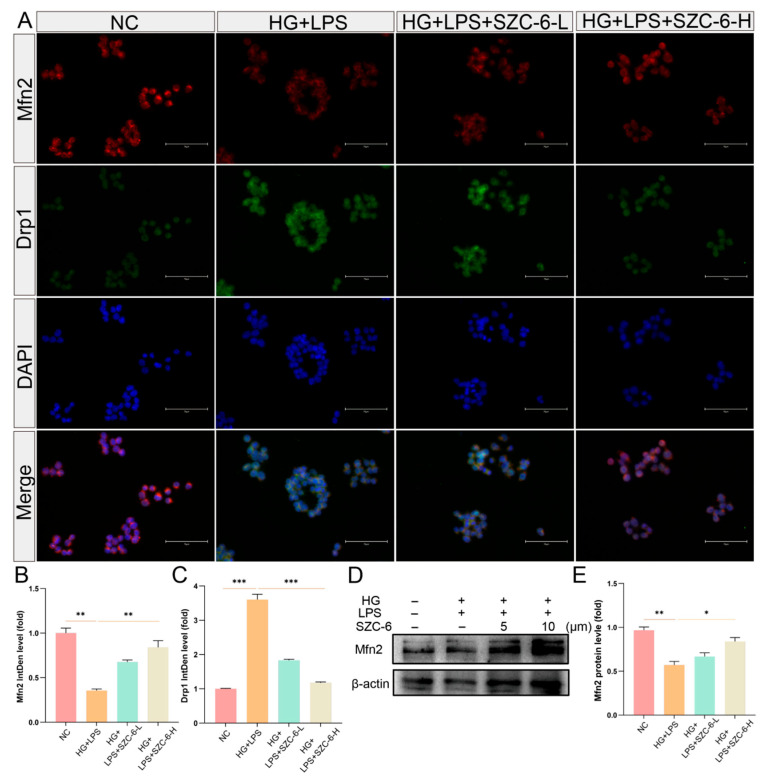
SZC-6 maintains mitochondrial functional homeostasis. (**A**) Immunofluorescence (IF) showing Drp1 and Mfn2 protein expression levels after LPS and LPS + SZC-6 treatment. Scale bar: 75 μm. (**B**,**C**) Statistical plots of Mfn2 and Drp1 fluorescence intensity. (**D**) Western blot (WB) detection of Mfn2 protein levels in each cell group (Figure S2). (**E**) Statistical plots of the grey value of Mfn2 protein bands for each group. Data are presented as mean ± SD (*n* = 3). * p < 0.05, ** p < 0.01, *** p < 0.001. NC refers to the negative control, LPS refers to the high-glucose model group, SZC-6-L denotes 5 μM SZC-6, and SZC-6-H denotes 10 μM SZC-6.

Mitochondrial dynamics play a critical role in various cellular processes, including oxidative stress and apoptosis [24]. In mammalian cells, mitochondrial fission is mediated by Drp1 and mitochondrial junction protein Fis1, while fusion of outer and inner mitochondrial membranes is regulated by mitophagy proteins (Mfn1, Mfn2) and optic atrophy 1 (OPA1) [25,26]. Initially, we assessed Mfn2 and Drp1 protein expression in each group using immunofluorescence. The model group received HG + LPS alone; experimental groups were treated with HG + LPS plus SZC-6-L (5 μmol/L) or SZC-6-H (10 μmol/L), with assays performed after 24 h. The Native Control (NC) group was cultured in complete DMEM only. We observed an upregulation of the mitochondrial fission protein Drp1 in the LPS group compared to the NC group. Following administration of low and high concentrations of SZC-6, Drp1 expression decreased while Mfn2 expression increased (Figure 3A–C). Subsequently, immunoblotting revealed increased Mfn2 protein levels with SZC-6 treatment compared to the LPS group (Figure 3D,E and Figure S2). These findings suggest that SZC-6 promotes favorable mitochondrial dynamics under conditions of cellular oxidative stress.

### 2.4. SZC-6 Inhibits M1 Macrophage Polarisation in a High-Glucose Inflammatory Environment In Vitro

**Figure 4:**
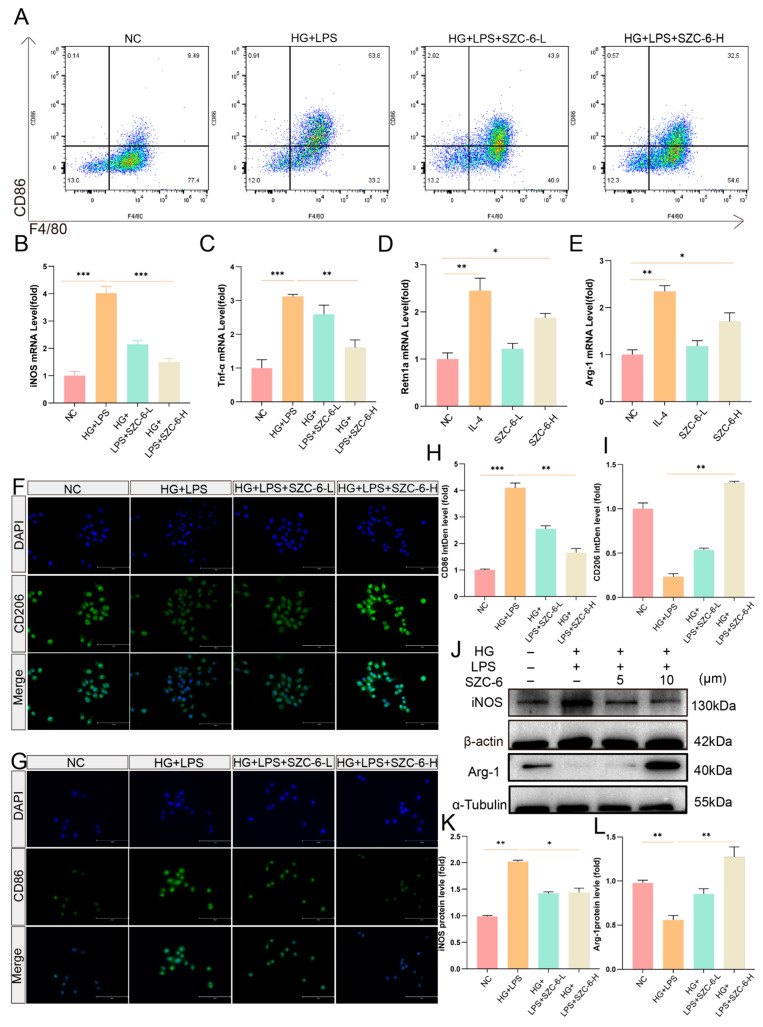
SZC-6 inhibits M1 polarization and reduces the M1/M2 ratio in vitro. (**A**) Flow cytometry was employed to determine the ratio of F4/80+/CD86+ in each group. (**B**–**E**) RT-qPCR was used to measure mRNA levels of *iNos*, *TNF-α*, *Arg-1*, and *Retnla*. (**F**,**G**) Representative immunofluorescence images of CD206 and CD86 after 24-h treatment with HG + LPS and HG + LPS + SZC-6 (L, H) (*n* = 3). Scale bar: 100 μm. (**H**,**I**) Quantitative statistical plots of immunofluorescence intensity of CD206 and CD86 proteins in each group (*n* = 3). (**J**) Western blot analysis (*n* = 3) showing iNos and Arg-1 protein expression levels after 24-h treatment with HG + LPS and HG + LPS + SZC-6 (L, H) (Figures S3 and S4). (**K**,**L**) Quantitative analysis of iNos and Arg-1 protein expression. Data are presented as means ± SD (*n* = 3). * p < 0.05, ** p < 0.01, *** p < 0.001. NC refers to negative control, LPS refers to the high-glucose model group, SZC-6-L denotes 5 μM SZC-6, and SZC-6-H denotes 10 μM SZC-6.

The sustained M1 pro-inflammatory polarization of macrophages is pivotal in the delayed healing of diabetic ulcers (DU) due to excessive accumulation of ROS [27]. Hyperglycemic inflammation models were induced by HG + LPS. Experimental cohorts received HG + LPS plus SZC-6-L (5 μmol/L) or SZC-6-H (10 μmol/L). The IL-4 group was supplemented with 20 ng/mL IL-4 in complete DMEM, while the SZC-6 monotherapy groups were exposed to compounds without IL-4, with assays performed after 24 h. The NC group received complete DMEM only. In flow cytometry experiments, we observed that the percentage of M1 macrophages (F4/80+/CD86+) in the LPS group increased from 9.49% to 53.8% compared to the NC group. Moreover, administration of a high dose of SZC-6 reduced the percentage of M1 macrophages in the SZC-6-H group from 53.8% to 32.5% compared to the LPS group (Figure 4A). RT-qPCR results revealed significant down-regulation of *iNos* and *TNF-α* following SZC-6 treatment (Figure 4B,C), while *Arg-1* and *Retnla* were significantly up-regulated (Figure 4D,E). We assessed protein expression levels of M1-associated markers (iNos, CD86) and M2-associated markers (Arg-1, CD206). Immunofluorescence revealed that LPS incubation decreased CD206 expression but increased CD86 levels in RAW264.7 cells. The administration of low and high doses of SZC-6 reversed this effect, enhancing CD206 expression and reducing CD86 levels compared to the LPS group (Figure 4F–I). Western blot analysis further confirmed that SZC-6 reduced iNos expression and increased Arg-1 expression levels compared to the LPS group (Figure 4J–L and Figures S3 and S4). These findings indicate that SZC-6 inhibits M1 polarization and reduces the M1/M2 ratio in vitro.

### 2.5. The Treatment with SZC-6 Accelerates Wound Healing in Diabetic Mice

**Figure 5:**
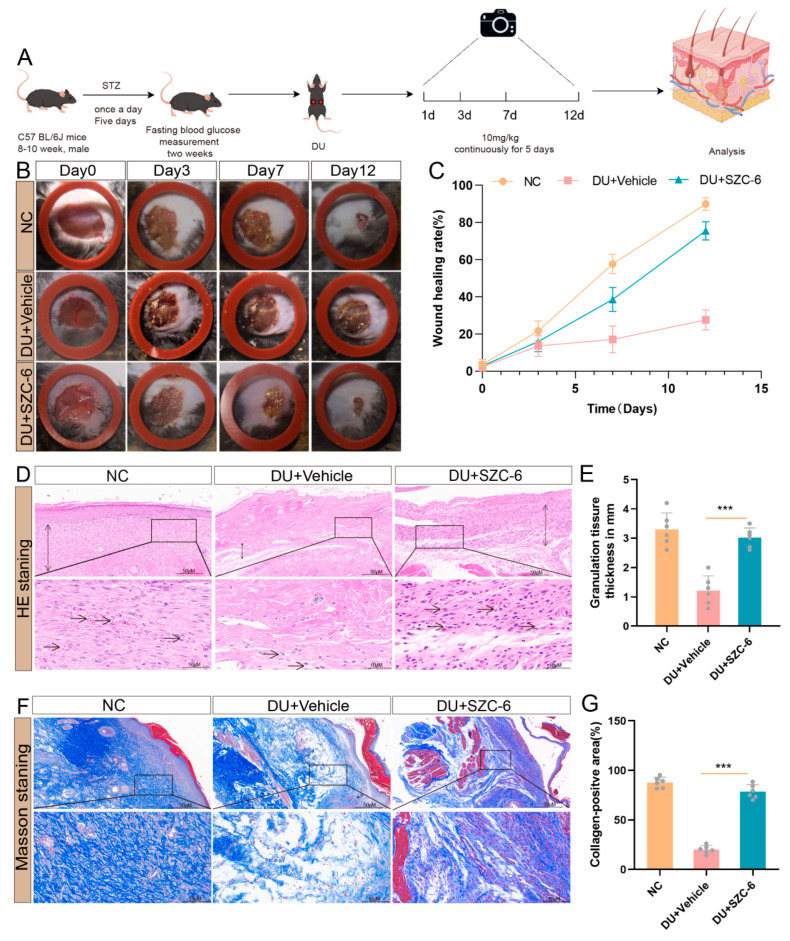
Treatment with SZC-6 improves wound healing in STZ-induced diabetic mice. (**A**) Schematic illustration of SZC-6 administration for diabetic wounds. (**B**) Representative images of diabetic scald wounds treated with the compound SZC-6 at each time point (the diameter of the ring inside the red circle is 1.5 cm). (**C**) Statistics of wound healing rates in diabetic scald wounds at each time point in each group (*n* = 6). (**D**,**E**) Representative images of diabetic scald wounds on day 9 with HE staining. (**F**,**G**) Representative images of Masson staining of diabetic scald wounds on day 9. Data are presented as means ± SD (*n* = 6). *** *p* < 0.001. NC refers to negative control.

Previous studies have shown that diabetic patients experience delayed wound healing due to persistent inflammation in the surrounding tissues [28]. Mice were randomly assigned to NC, DU + Vehicle, and DU + SZC-6 groups. Diabetes was induced by STZ injection (Figure S5), followed by dorsal wound creation to establish the DU model. The DU + SZC-6 group received daily SZC-6 for five consecutive days, while the NC and DU + Vehicle groups were administered equivalent volumes of physiological saline. Our results demonstrated that the DU group exhibited significantly prolonged wound healing and lower wound healing rates compared to the NC group (Figure 5A,B). Treatment with SZC-6 accelerated wound healing compared to the DU group. The difference in wound healing was more pronounced from the seventh day onward and persisted throughout the healing process (Figure 5C). HE staining on day 9 revealed increased granulation tissue formation in the SZC-6-treated groups compared to the DU group (Figure 5D,E). Masson’s staining further illustrated that collagen fibers in the wound tissue of DU mice were irregularly arranged with reduced collagen deposition compared to the NC group. In contrast, SZC-6 administration enhanced collagen deposition, resulting in a more regular and parallel arrangement of collagen fibers on the skin surface (Figure 5F,G).

### 2.6. The Treatment with SZC-6 Reduces the M1/M2 Ratio in the Wound Tissue of Diabetic Mice

**Figure 6:**
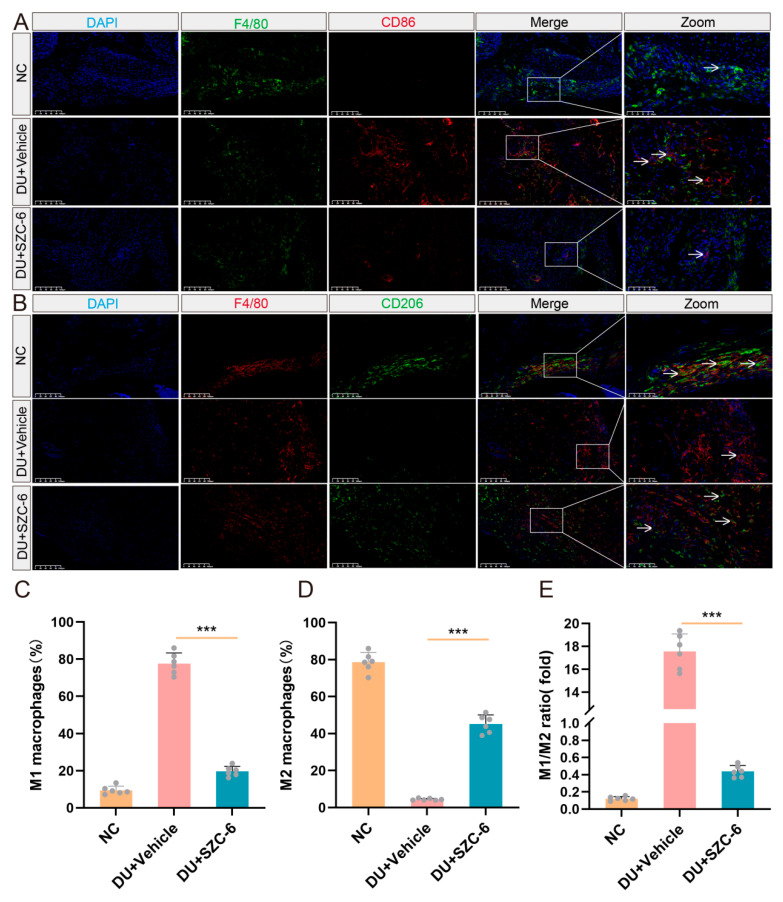
SZC-6 reduces the M1/M2 ratio in diabetic mice. (**A**) Representative images of CD86 immunofluorescence in diabetic scald wounds on day nine. (**B**) Representative images of CD206 immunofluorescence in diabetic scald wounds on day nine. Scale bar of Merge = 100 μm, scale bar of Zoom = 50 μm. (**C**–**E**) We quantified the proportion of M1 and M2 macrophages by counting CD86- and CD206-positive nuclei, expressed as a percentage of total F4/80-positive. Data are presented as means ± SD (*n* = 6). *** p < 0.001. NC refers to negative control.

Macrophages play a crucial role in wound healing, and hyperactivation of pro-inflammatory M1 macrophages can delay wound healing [29]. The DU model was established by STZ injection and dorsal wounds. The DU + SZC-6 group received SZC-6 for five days, while the NC and DU + Vehicle groups received isovolumetric normal saline. We identified DU M1-like macrophages using F4/80+/CD86+. Immunofluorescence findings revealed that, compared to the NC group, the DU group still had a significant number of M1-like macrophage clusters on day nine. However, this number decreased after SZC-6 administration (Figure 6A). Immunofluorescence results for CD206 also demonstrated that SZC-6 treatment significantly increased the population of M2-like macrophages in the traumatized tissues compared to the DU group (Figure 6B). Subsequently, we quantified the stained sections of M1 and M2, respectively (Figure 6C–E). In summary, our study revealed that SZC-6 may accelerate diabetic wound healing by modulating the M1/M2 ratio.

### 2.7. SZC-6-Induced Changes in the NF-χB Signaling Pathway in LPS-Treated RAW264.7 Cells

**Figure 7:**
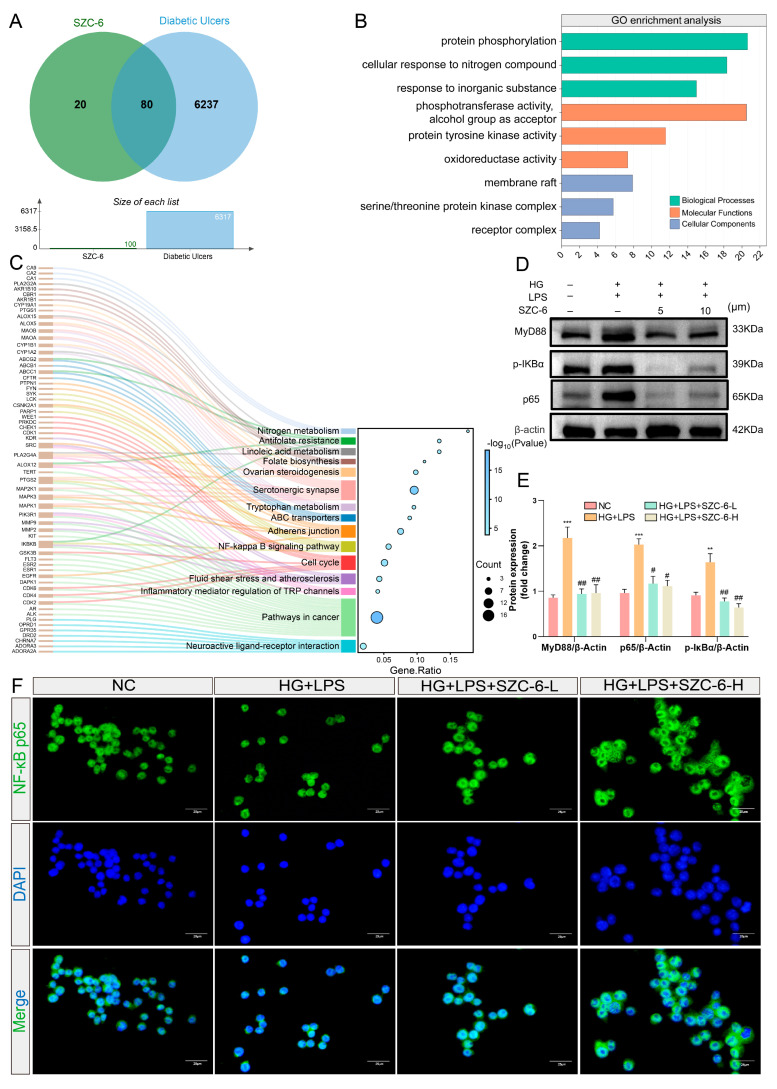
SZC-6 Modulation of the NF-χB pathway in LPS-treated RAW264.7 cells. (**A**) Venn diagram showing overlapping potential compound targets of SZC-6 and therapeutic targets for DU treatment. (**B**) Bubble plot of GO function analysis of potential compound targets of SZC-6 in DU treatment. (**C**) Bubble plot of KEGG enrichment of potential compound targets of SZC-6 in DU treatment. (**D**,**E**) Effects of SZC-6 at different doses on MyD88, p-IκBα, and p65 expression in LPS-treated RAW264.7 cells (Figure S6). (**F**) Effects of SZC-6 at different doses on the nuclear translocation of p65 in LPS-treated RAW264.7 cells. Scale bar = 25 μm. Data are presented as Mean ± SEM, *n* = 3, ** p < 0.01, and *** p < 0.001 indicate significant differences compared to the NC group, while ^#^ *p* < 0.05 and *^##^ p* < 0.01 indicate significant differences compared to the HG + LPS group. NC refers to negative control, HG + LPS refers to the high-glucose model group, SZC-6-L denotes 5 μM SZC-6, and SZC-6-H denotes 10 μM SZC-6.

To investigate the molecular mechanism of SZC-6 in diabetic wound healing treatment, a network pharmacology analysis based on SZC-6’s chemical components was conducted. One hundred potential targeting compounds in SZC-6 were identified using the SwissTargetPrediction database. Additionally, 6317 potential therapeutic targets of DU were identified via the GeneCards, HERB, DisGeNET, and OMIM databases (Supplementary Files-B). Notably, 80 overlapping targets were found between SZC-6’s potential compounds and DU’s potential therapeutic targets, suggesting their role in SZC-6’s effect on DU treatment (Figure 7A). GO functional and KEGG pathway enrichment analyses with these 80 core targets revealed that SZC-6 affects various biological processes, cellular components, and molecular functions, including positive regulation of phosphorylation, receptor complex function, and protein tyrosine kinase activity (Figure 7B). KEGG enrichment analysis indicated that SZC-6 might treat DU by modulating the NF-χB signaling pathway (Figure 7C). Cellular-level validation was performed to verify KEGG enrichment findings. The HG + LPS model was induced with co-treatment of 30 mmol/L of HG and 1 μg/mL of LPS. Experimental groups received co-treatment with SZC-6-L (5 μM) or SZC-6-H (10 μM) for 24-h incubation. Western blotting demonstrated that SZC-6 significantly reduced the expression of MyD88, IκBα, and NF-χB p65 in LPS-treated RAW264.7 cells (Figure 7D,E and Figure S6). Furthermore, immunofluorescence results showed LPS-induced noticeable nuclear translocation of NF-χB p65, decreased by SZC-6 treatment (Figure 7F), suggesting SZC-6’s potential role in DU treatment via the NF-χB inflammatory signaling pathway.

## 3. Discussion

Diabetic wounds place a heavy burden on the healthcare system, and despite the range of treatment options available, the outcomes remain quite limited [30]. In diabetic patients, hyperglycemia leads to a persistent predominance of pro-inflammatory M1 macrophages at the wound site, hindering the healing process [31]. Dysregulation of macrophage polarization in diabetic wound healing has become a prominent research focus.

Our study demonstrates that SZC-6, a SIRT3 agonist, effectively promotes diabetic wound healing by inhibiting M1-like macrophage polarization. In vitro experiments revealed that SZC-6 decreased inflammatory factors and improved the oxidative stress state while rebalancing mitochondrial function. In animal experiments, SZC-6-treated wounds showed improved tissue formation, reduced pro-inflammatory M1 macrophage presence, and increased M2 macrophage proportion. These results suggest that SZC-6 may accelerate diabetic wound healing by modulating the M1/M2 ratio.

Diabetic wound healing represents a complex process where the inflammatory response assumes a pivotal role. SIRT3, predominantly situated in mitochondria, emerges as a promising therapeutic target for inflammation-related diseases [32,33]. Elevated levels of reactive oxygen species (ROS) instigate oxidative stress, exacerbated by mitochondrial ROS stemming from the electron transport chain (ETC) [34]. Li et al. demonstrated that ROS overproduction activates the NF-χB/NLRP3 pathway, promotes the expression of inflammatory factors, and exacerbates the oxidative stress state [35]. The primary activation mechanisms of the NF-χB pathway involve NF-χB p65 activity and nuclear translocation [36]. Several studies have shown that reducing the excessive accumulation of intracellular ROS inhibits the expression of nuclear NF-χB p65, effectively controlling inflammation [37,38,39].

Our study reveals SZC-6’s significant enhancement of the mitochondrial fusion protein Mfn2, attenuation of intracellular and mitochondrial ROS generation, and reduction in nuclear NF-χB p65 expression. Notably, macrophage plasticity is a salient feature, with a recent study by Hu et al. unveiling that NF-χB inhibition thwarts M1-like macrophage polarization [40]. Previous studies on coumarin and its derivatives have demonstrated anti-inflammatory effects through NF-χB signaling suppression [41]. In vitro outcomes indicate that the coumarin analog SZC-6 curbs NF-χB activation by reducing IκBα phosphorylation and the expression of NF-χB p65 protein, inhibiting M1-like macrophage polarization and promoting M2-like macrophage polarization.

Our findings suggest that SZC-6 may have a potential role in diabetic wound healing by modulating macrophage polarization via NF-χB signaling suppression. It remains to be explored whether there are other mechanisms by which SZC-6 acts as a SIRT3 agonist in the treatment of diabetic ulcers.

## 4. Materials and Methods

### 4.1. Animal Treatment

The animal models used in this experiment were mice. The study is reported in accordance with ARRIVE guidelines. All animal experiments were approved by the Animal Ethics Committee of Guangdong Pharmaceutical University (ethics number: GDPULACSPF2022208), and all methods were performed in accordance with the relevant guidelines and regulations. All animal studies were performed following the National Institutes of Health (NIH) guide for the Care and Use of Laboratory Animals. In total, 30 Male C57/B6 mice (weight 19–23 g, 6–8 weeks old) were purchased from Guangdong Pharmachem Biotechnology Co., Ltd. (Dongguan, China) and were kept in a pathogen-free environment at 21–23 °C with a 12-h daylight/dark cycle. After a week of acclimatization feeding, the mice were randomly divided into three groups: normal (NC), diabetic ulcer (DU), and diabetic ulcer + SZC-6 (SZC-6) groups (8 mice per group). Following 12 h of a fasting period, diabetes was induced in mice in the diabetic and treatment groups by intraperitoneal injection of freshly prepared STZ (60 mg/kg/d) in a sodium citrate buffer solution (pH = 4.5), and the control group by injection of an equal amount of sodium citrate buffer. Fasting blood glucose was measured weekly using a glucometer, and mice with blood glucose levels > 11.7 mmol/L for more than 2 weeks were considered successfully modeled. A total of 22 mice were used for diabetes modeling, and 18 mice were successfully modelled, giving a model success rate of 81%. Two total excision wounds with a diameter of 8 mm were then created on the dorsal side of the mice. The treatment group was injected with 100 μL of SZC-6 (10 mg/kg) via intraperitoneal injection on days 0, 1, 2, 3, and 4 post-injuries; mice in the NC and DU groups were injected intraperitoneally with an equal normal saline as a control. Images of the wound area were taken on postoperative days 0, 7, and 12 and analyzed using ImageJ software v1.8.0.112 (National Institutes of Health, Bethesda, MD, USA). The skin around the wounds of four mice in each group was collected on days 9 and 12, respectively, for subsequent experiments. The wound area was measured with Image J image analysis software, and wound sites were harvested on day 9 for pathological analysis. The wound healing rates were calculated as (wound area on day 0 wound area on day N)/wound area on day 0 × 100%. At the end of the experiment, all animals were euthanized by a lethal dose of sodium pentobarbital (80 mg/kg BW) and euthanized by cervical dislocation.

### 4.2. Cell Culture and Processing

RAW264.7 cells were incubated in DMEM (Gibco, Grand Island, NY, USA) supplemented with 10% FBS (Oricell, Shanghai, China), 100 U/mL of penicillin, and 100 U/mL of streptomycin (penicillin-streptomycin, Biosharp, Hefei, China) at 37 °C with 5% CO_2_. The cells were divided into four main groups: NC (containing 5.5 mM D-glucose), HG (high glucose concentration, 30 mM) + LPS (1 μg/mL, 24 h), HG + LPS + SZC-6-L (5 μM), and HG + LPS + SZC-6-H (10 μM).

### 4.3. CCK-8 Cytotoxic Assay

We used the CCK-8 assay to determine the activity of RAW264.7 cells (5 × 10^3^ cells/well) at various concentrations of SZC-6. The RAW264.7 cells were placed into 96-well plates and then exposed to SZC-6 at concentrations of 0, 10, 15, 20, 30, 40, and 50 μM for 24 h, with the 0 concentration serving as a control. Afterward, the cells were incubated with CCK-8 working solution for 2 h at 37 °C. The absorbance at 450 nm was then measured using a spectrophotometer (Thermo, Waltham, MA, USA). Cell viability was calculated by comparing the absorbance ratio of each treatment group to the control group.

### 4.4. HE and Masson Staining

The peripheral tissues of the wound were fixed in paraformaldehyde (4%), followed by standard histologic procedures and fixation embedding. Next, 4 μm-thick tissue sections were prepared and subjected to HE and Masson staining. In addition, the histological wound healing scores were calculated by assessing indicators such as granulation tissue thickness and remodeling in each mouse.

### 4.5. Immunofluorescence

A 4 μm-thick layer of paraffin-coated tissue was cut and washed in distilled water for 10 min. To minimize non-specific reactions, the tissues were closed in 5% normal serum—0.25% Triton X-100 in PBS solution for 1 h at room temperature. To analyze M1 macrophages and M2 macrophages, the tissues were incubated overnight at 4 °C with anti-CD206(HA722892; 1:1000; HUABIO, Hangzhou, China); anti-CD86(ER1906–01, 1:1000; HUABIO, Hangzhou, China); and F4/80(HA721745, 1:1000; HUABIO, Hangzhou, China). After rinsing 3 times with PBS for 5 min each, the tissues were exposed to Alexa Fluor 488-labeled goat anti-rabbit antibody (1:1000) and Cy-7-labeled goat anti-mouse (1:1000) antibody for 1 h at room temperature. Cell nuclei were stained using 4′,6-diamidino-2-phenylindole (DAPI) followed by a fluorescence microscope.

RAW264.7 cells were cultured in 12-well plates and subjected to the indicated treatments for 24 h. After fixation in paraformaldehyde (4%) for 30 min, the cells were rinsed with PBS three times for 5 min each, and then exposed to 0.1% Triton-X-100 in phosphate buffered saline with Tween 20 (PBST) for 15 min at room temperature. Following a 1-h incubation with 5% BSA, the cells were incubated with the primary antibody at 4 °C overnight. After rinsing three times with PBST for 5 min each, the cells were exposed to a fluorescent secondary antibody for 1 h at room temperature. DAPI staining was performed in the dark for 3 min, followed by three rinses with PBST for 5 min each. The stained cells were examined under a fluorescence microscope after sealing the slides.

### 4.6. RT-qPCR Assay

To perform the RT-qPCR assay, the total RNA was extracted from RAW264.7 cells using Vazyme biotech reagents (Vazyme biotech, Nanjing, China). The cDNA synthesis was performed by a reverse transcription kit with 1 μg of total RNA. β-Actin served as a housekeeping gene. The primer sequences for each target gene can be found in the supplementary information (Table S1). Power SYBR Green PCR premix was utilized for the RT-qPCR, which was conducted on an ABI Viia 7 detector system following standard procedures. The relative mRNA expression was determined using the 2^−∆∆CT^ method, with β-actin used for normalization.

### 4.7. Western Blot

We utilized a BCA kit (P0010, Beyotime, Shanghai, China) to determine protein levels in cell samples and tissue homogenates. Proteins were separated using a 10% SDS-PAGE gel, and membranes with nonspecific binding sites (Millipore, Burlington, MA, USA) were blocked with 5% skimmed milk powder at room temperature for 2 h. The PVDF membranes were then probed with the specific primary antibodies, including antibodies for Arg-1 (A1847,1:1000, Abclonal, Wuhan, China), iNos (ER1706−89, 1:1000, HUABIO, Hangzhou, China), tubulin (AF5001,1:1000, Beyotime, Shanghai, China), β-actin (49,294, 1:1000; Signalway Antibody, Greenbelt, MD, USA), MyD88 (GB111554, 1:1000; Servicebio Technology, Wuhan, China), NF-χB p65 (21,011, 1:500; Signalway Antibody, Greenbelt, MD, USA), and Phospho-IκB (ET1609–78, 1:2000; HUABIO, Hangzhou, China), for more than 12 h. Subsequently, the PVDF membranes were incubated with HRP-conjugated secondary antibodies, and bands were analyzed using a chemiluminescence method.

### 4.8. Flow Cytometric Analysis

RAW264.7 cells were treated with a range of concentrations of LPS or SZC-6 for 24 h. After the cells were collected, Fc receptors were blocked using TruStainFcX PLUS (anti-CD16/32, BioLegend, San Diego, CA, USA). Cell staining with mAbs for the mouse surface markers CD86 and F4/80 was performed. Relevant mAbs were stained at 4 °C for 30 min. The cells were washed three times with Cell Staining Buffer (BioLegend) before analysis using FACS Calibur (BD Biosciences, San Jose, CA, USA) and CellQuest v3.3 software (BD Biosciences, San Jose, CA, USA).

### 4.9. JC-1 Staining

The RAW264.7 cells were seeded in 12-well plates (1 × 10^5^ cells/well) and were stained with JC-1 following the manufacturer’s instructions of the JC-1 staining assay kit (M8650, Solarbio, Beijing, China). In brief, after interventions, cultured RAW264.7 cells were washed with 1 × PBS 3 times and stained with the JC-1 staining working solution for 20 min at 37 °C. After incubation, the supernatant was removed and washed twice with JC-1 staining buffer. Then, the absorption of JC-1 monomer (fluorescence excitation set at 490 nm; fluorescence emission set at 530 nm) and JC-1 aggregates (fluorescence excitation 525 nm; fluorescence emission 590 nm) was observed under an inverted fluorescence microscope (Thermo, Waltham, MA, USA). Red and green fluorescence corresponded to JC-1 aggregates and monomers; the mitochondrial membrane potential difference between groups was reflected by the red/green fluorescence ratio (aggregates/monomers).

### 4.10. ROS Detection

RAW264.7 cells were seeded in 12-well plates (1 × 10^5^ cells/well). DCFH-DA was added to the cells at a concentration of 2 μM after LPS and/or SZC-6 treatment, and the cells were incubated for 30 min. Production of ROS was measured under a fluorescence microscope (Zeiss Fluorescence Microscope, Oberkochen, Germany).

### 4.11. Network Pharmacology

The potential targets of SZC-6 were predicted via the SwissTargetPrediction database (http://www.swisstargetprediction.ch/, accessed on 3 July 2024). The DU therapeutic targets were collected from the GeneCards (https://www.genecards.org/, accessed on 3 July 2024), HERB (http://herb.ac.cn, accessed on 3 July 2024), DisGeNET (mailto: https://www.disgenet.org/, accessed on 3 July 2024), and OMIM (mailto: https://www.omim.org/, accessed on 3 July 2024) databases using the keywords “diabetic foot ulcer”. We combined these targets with the potential targets of SZC-6. GO and KEGG analyses were performed for the overlapping genes using the Metascape Database (https://metascape.org/gp/index.html#/main/step1, accessed on 4 July 2024).

### 4.12. Statistical Analysis

Statistical analysis was conducted using Prism software (version 8.0; Prism Software Corp). The results of each experiment were presented as the mean ± SD of three replicates. Independent two-tailed Student’s *t*-tests were used for comparisons between the two groups. Multiple comparisons were performed using one-way or two-way analysis of variance (ANOVA). *p* values < 0.05 were considered statistically significant. The datasets generated and analyzed during the current study are available in the [figshare] repository, [10.6084/m9.figshare.27628626].

## 5. Conclusions

This study introduced a new SIRT3 activator called SZC-6, which was developed based on the crystal structure of the MnsodK68AcK-SIRT3 complex by modifying C12 as a lead compound. Our study showed that SZC-6 reduces ROS levels, enhances mitochondrial membrane potential, decreases the proportion of M1 macrophages, and inhibits the activation of the NF-χB pathway. In experiments conducted in live organisms, SZC-6 treatment accelerated wound healing in diabetic mice. Although SIRT3 agonists are known for their anti-inflammatory and ROS-reducing properties in diabetic wound healing, our study uniquely demonstrated SZC-6’s ability to modulate macrophage polarization via SIRT3 activation, specifically inhibiting the pro-inflammatory M1-like polarization in diabetic wound tissue and thereby accelerating wound healing. The study concluded that SZC-6 inhibited the activation of the NF-χB pathway, thus reducing the inflammatory response and promoting skin healing in diabetic ulcers. SZC-6 provides valuable insights into potential therapeutic approaches for improving diabetic wound healing.

## Data Availability

The datasets generated during and/or analyzed during the current study are available. All data have been deposited in the Figshare database with DOI: 10.6084/m9.figshare.27628626. The remaining data are presented in the Supplementary Materials. The raw data supporting the conclusions of this article will be made available by the authors (Ang Xuan) on request.

## Supplementary Materials

The following supporting information can be downloaded at: https://www.mdpi.com/article/10.3390/ph18081143/s1, Supplementary Files-A: Figure S1: Cell viabilities of RAW264.7 cells treated with SZC-6 at different concentrations; Figure S2: Western blot analysis of β-actin, Mfn2 expression in the RAW264.7 cell (n = 3); Figure S3: Western blot analysis of α-Tubulin, Arg-1 expression in the RAW264.7 cell (n = 3); Figure S4: Western blot analysis of iNos, β-Actin expression in the RAW264.7 cell (n = 3); Figure S5: Establishment of diabetic wound model; Figure S6: Western blot analysis of β-actin, NF-χB p65, MyD88, p-IκBα expression in the RAW264.7 cell (n = 3); Table S1: The primer sequences used in the quantitative real-time. Supplementary Files-B: Table S2: Drug Targets; Table S3: Disease Targets; Table S4: Intersecting targets; Table S5: BP; Table S6: MF; Table S7: CC; Table S8: KEGG.

## Abbreviations

C12, 7-Hydroxy-3-(4-methoxyphenyl)-2H-benzopyran-2-one; DU, diabetic ulcers; ETC, electron transport chain; HST, hesperidin; M1, activated macrophages; M2, selectively activated macrophages; PBST, phosphate-buffered saline supplementation; ROS, reactive oxygen species; SIRT3, Sirtuin 3; SZC-6, 3-(2-bromo-4-hydroxyphenyl)-7-hydroxy-2H-chromen-2-one; STZ, Streptozotocin; Δψm, mitochondrial membrane potential.
