# Supplementary material for: SZC-6 Promotes Diabetic Wound Healing in Mice by Modulating the M1/M2 Macrophage Ratio and Inhibiting the MyD88/NF-χB Pathway
Source: Pharmaceuticals (Basel). 2025 Jul 31;18(8):1143. doi: 10.3390/ph18081143 (PMC12389266; doi:10.3390/ph18081143)
Supplement: Supplementary file 1 [file pharmaceuticals-18-01143-s001.zip › Supplementary files-A.pdf]

Supporting Information

SZC-6 Promotes Diabetic Wound Healing in Mice by Modulating M1/M2 Macrophage Ratio and Inhibiting MyD88/NF-κB Pathway

Supplementary Figures and Tables

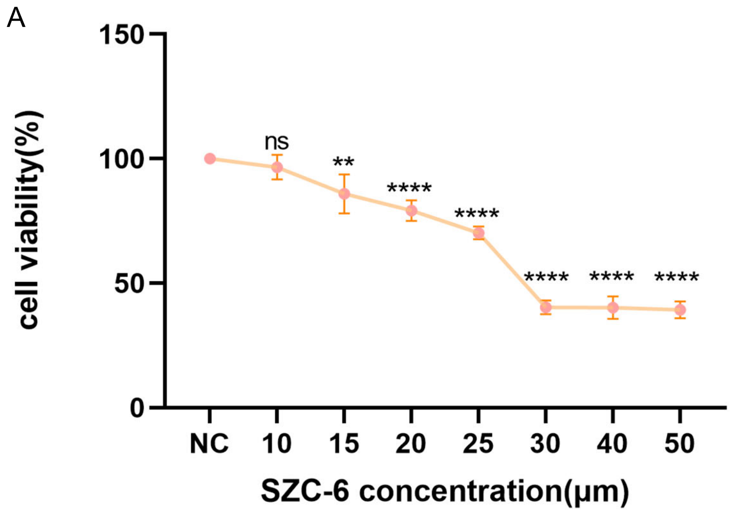

Figure. S1. Cell viabilities of RAW264.7 cells treated with SZC-6 at different concentrations.

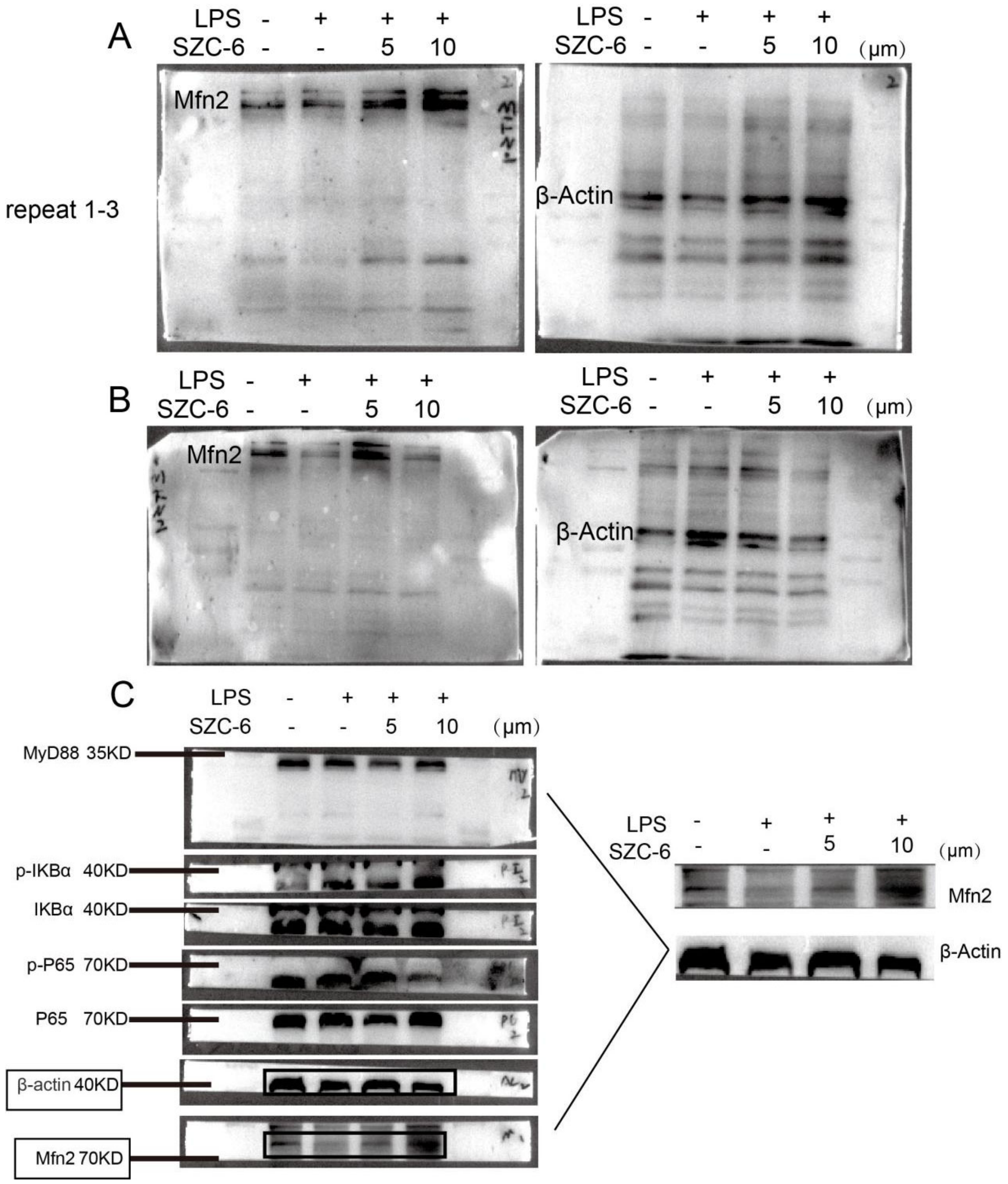

Figure. S2. Western blot analysis of β-actin, Mfn2 expression in the RAW264.7 cell. (**A-C**) The A-C panels depict tri-plicate WB analyses of β-actin and Mfn2, demonstrating that S-ZC-6 upregulates Mfn2 protein expression relative to HG+LPScontrols (*n* = 3).

repeat 1-3

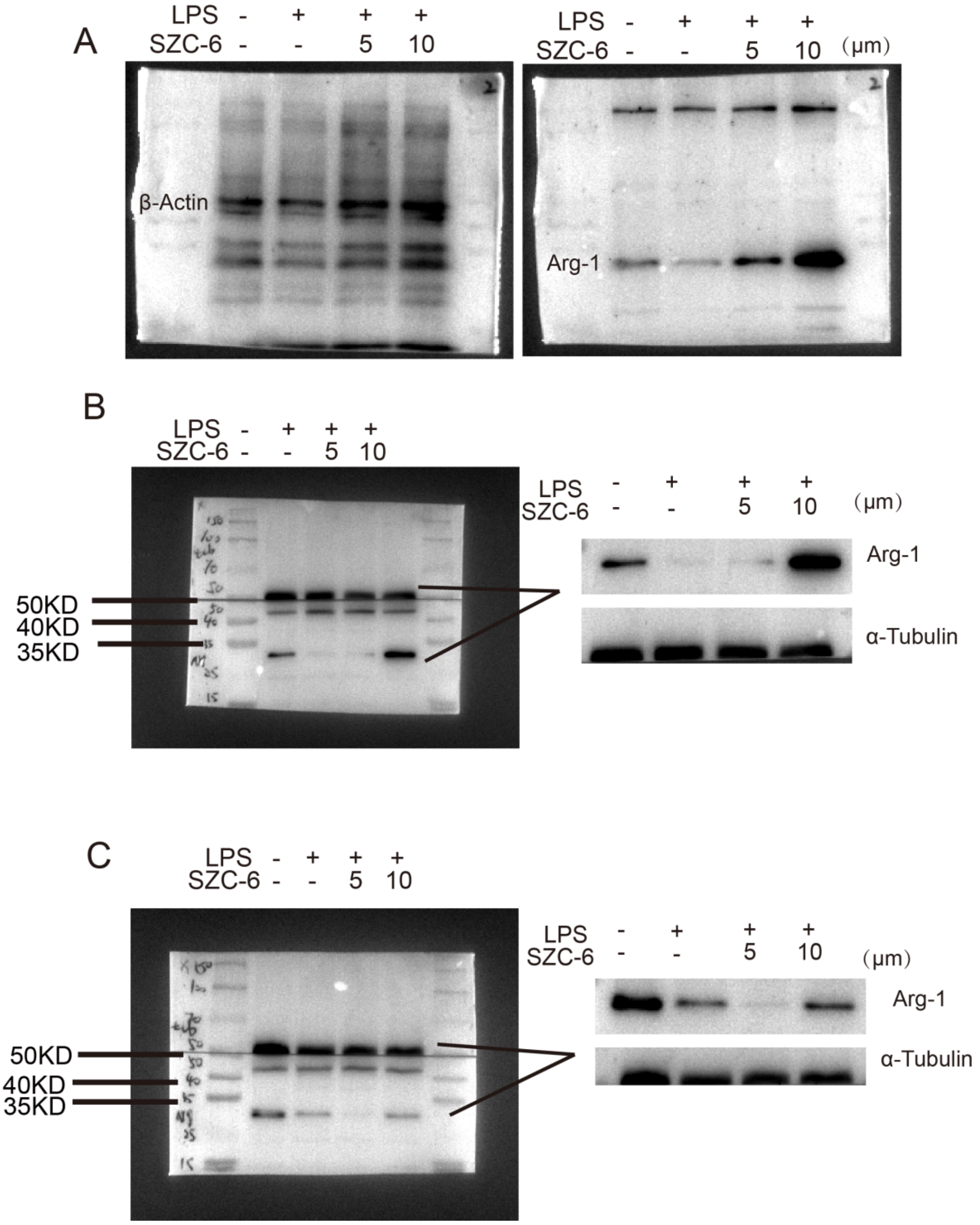

Figure. S3. Western blot analysis of α-Tubulin, Arg-1 expression in the RAW264.7 cell. (**A-C**) The A-C panels depict triplicate WB analyses of α-Tubulin and Arg-1 demonstrating t-hat SZC-6 upregulates Arg-1 protein expression relative to H-G+LPS controls (*n* = 3).

iNOS repeat A-B

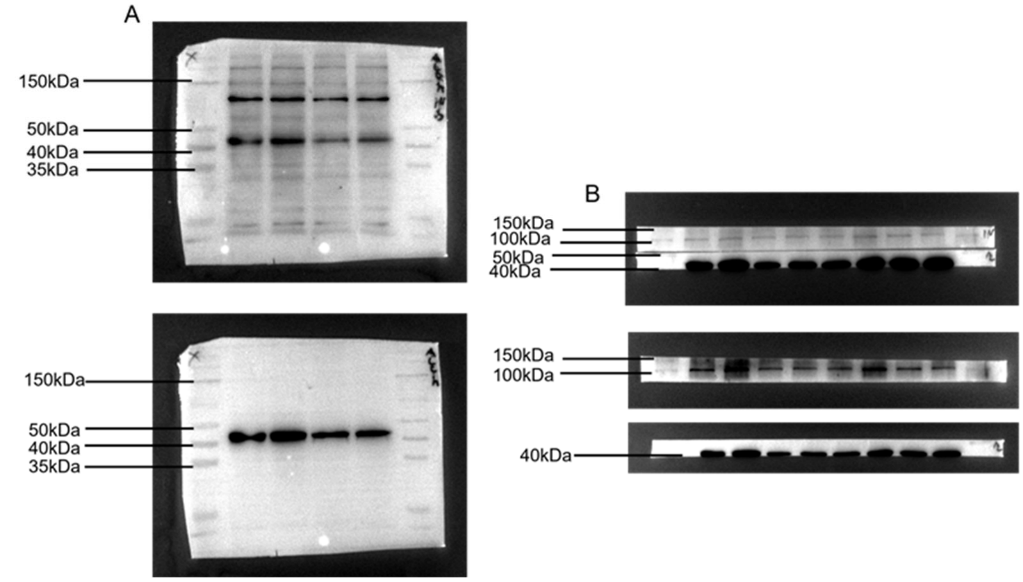

Figure. S4. Western blot analysis of iNos, β-Actin expression in the RAW264.7 cell. (**A-C**) The A-C panels depict tripl-icate WB analyses of iNos and β-Actin demonstrating that SZ-C-6 reduces iNos protein expression relative to HG+LPS contr-ols (n = 3).

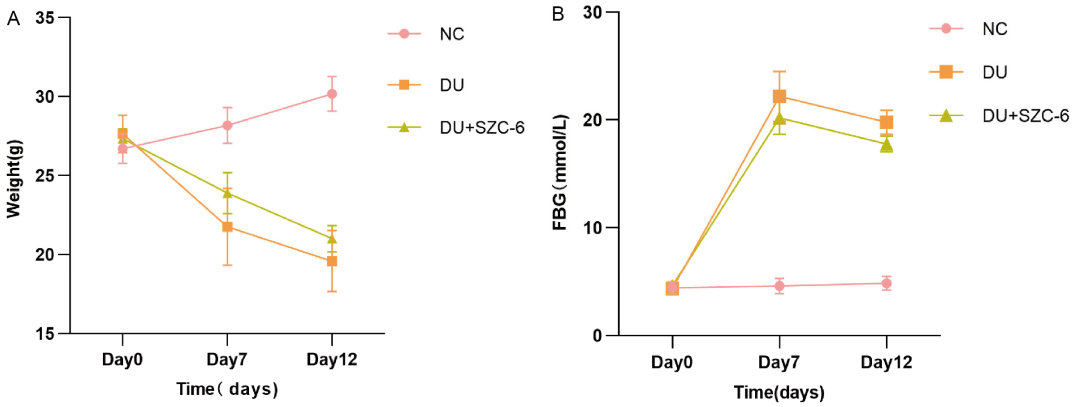

Figure. S5. Establishment of diabetic wound model.

(A) body weight level, SZC-6 treatment increased body weight in diabetic ulcer mice versus untreated controls. (B) blood gluc -ose levelof mice, SZC-6 treatment significantly reduced fasting blood glucose levels in diabetic ulcer mice versus untreated c-ontrols (*n* = 6).

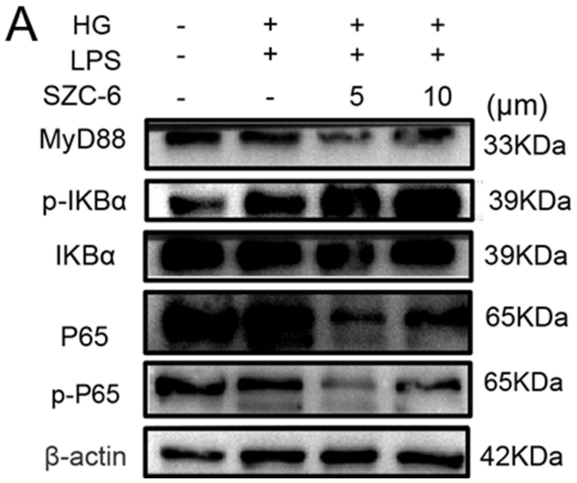

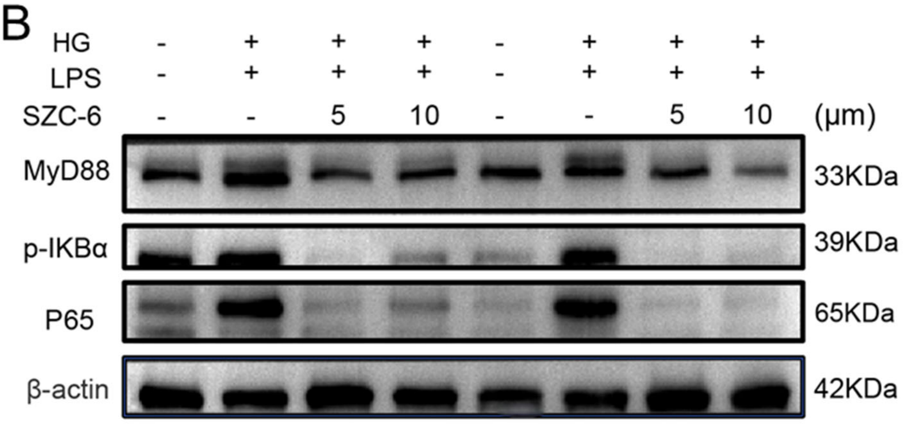

Figure. S6. Western blot analysis of β-actin,NF-χB p65, MyD88, p-IκBα expression in the RAW264.7 cell. (**A-C**) The A-C pane-ls depict triplicate WB analyses of β-actin,NF-χB p65, MyD88, p-IκBα demonstrating that SZC-6 reduces NF-χB p65, MyD88, p-IκBα protein expression relative to HG+LPS controls (*n* = 3).

Table S1. The primer sequences used in the quantitative real-time PCR experiments.

Gene name	forward primer（5'→3'）	reverse primer（5'→3'）
β-actin	TGACAGGATGCAGAAGGAGA	GCTGGAAGGTGGACAGTGAG
Arginase-1	CTCCAAGCCAAAGTCCTTAGAG	AGGAGCTGTCATTAGGGACATC
Retnla	CTCCACTGTAACGAAGACTC	GCAGTGGTCCAGTCAACGA
Il-6	CAACGATGATGCACTTGCAGA	TGTGACTCCAGCTTATCTCTTGG
Tnf-α	CTCAGCGAGGACAGCAAGG	AGGGACAGAACCTGCCTGG
iNos	GCGCTCTAGTGAAGCAAAGC	AGTGAAATCCGATGTGGCCTG
